# Bayesian and Maximum-Likelihood Modeling and Higher-Level Scores of Interpersonal Problems With Circumplex Structure

**DOI:** 10.3389/fpsyg.2021.761378

**Published:** 2021-10-29

**Authors:** Anneke C. Weide, Vera Scheuble, André Beauducel

**Affiliations:** Department of Methods and Diagnostics, Institute of Psychology, University of Bonn, Bonn, Germany

**Keywords:** inventory of interpersonal problems, interpersonal circumplex, confirmatory factor analysis, Bayesian structural equation modeling, regression factor scores, weighted sum scores, Big Five, grandiose narcissism

## Abstract

Difficulties in interpersonal behavior are often measured by the circumplex-based Inventory of Interpersonal Problems. Its eight scales can be represented by a three-factor structure with two circumplex factors, Dominance and Love, and a general problem factor, Distress. Bayesian confirmatory factor analysis is well-suited to evaluate the higher-level structure of interpersonal problems because circumplex loading priors allow for data-driven adjustments and a more flexible investigation of the ideal circumplex pattern than conventional maximum likelihood confirmatory factor analysis. Using a non-clinical sample from an online questionnaire study (*N* = 822), we replicated the three-factor structure of the IIP by maximum likelihood and Bayesian confirmatory factor analysis and found great proximity of the Bayesian loadings to perfect circumplexity. We found additional support for the validity of the three-factor model of the IIP by including external criteria-Agreeableness, Extraversion, and Neuroticism from the Big Five and subclinical grandiose narcissism-in the analysis. We also investigated higher-level scores for Dominance, Love, and Distress using traditional regression factor scores and weighted sum scores. We found excellent reliability (with *R*_*tt*_ ≥ 0.90) for Dominance, Love, and Distress for the two scoring methods. We found high congruence of the higher-level scores with the underlying factors and good circumplex properties of the scoring models. The correlational pattern with the external measures was in line with theoretical expectations and similar to the results from the factor analysis. We encourage the use of Bayesian modeling when dealing with circumplex structure and recommend the use of higher-level scores for interpersonal problems as parsimonious, reliable, and valid measures.

## Introduction

The Inventory of Interpersonal Problems (IIP) is one of the most widely used measures of difficulties in the interaction with other people (Horowitz et al., 1988, 2017). Interpersonal problems measured by the IIP have been linked to a variety of concepts in personality research, such as the Big Five (Nysæter et al., 2009), narcissism (Dickinson and Pincus, 2003; Ogrodniczuk et al., 2009), or the ability to identify and describe emotions (Weinryb et al., 1996). Clinical studies have shown that the IIP scales can be used to classify certain psychological disorders (Alden and Phillips, 1990; Pincus and Wiggins, 1990) and to evaluate treatment outcome of psychotherapy (Horowitz et al., 1988; Ruiz et al., 2004). In addition to its clinical applications, the IIP can also be used in non-clinical couple or family counseling, where interpersonal problems are of great relevance as well (Horowitz et al., 2017).

**Figure 1:**
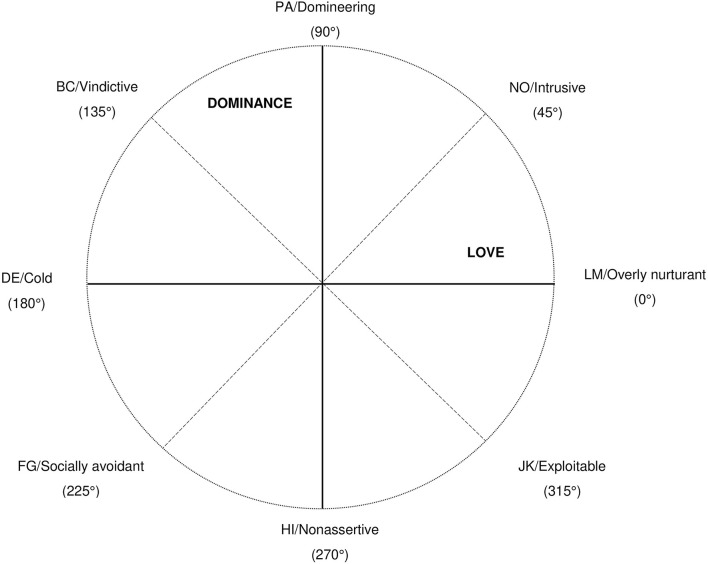
Circumplex structure and scales of the inventory of interpersonal problems (see also Gurtman, 1993).

The IIP in its current form has been developed in accordance with the interpersonal circumplex (Alden et al., 1990; Gurtman, 1993; Horowitz et al., 2017). Hence, it is supposed to fulfill circumplex structure with eight scales that are evenly distributed in 45° angular displacements (see Figure 1). The scales are LM/Overly nurturant (0°), NO/Intrusive (45°), PA/Domineering (90°), BC/Vindictive (135°), DE/Cold (180°), FG/Socially avoidant (225°), HI/Nonassertive (270°), and JK/Exploitable (315°). Circumplex structure can be specified and analyzed by a variety of models and procedures that differ in the assumptions and constraints imposed on the data (Gurtman and Pincus, 2000). The circumplex structure of the IIP can be modeled directly for the interrelations of the IIP octants. For example, the spatial representation model projects the spatial structure of the scales on a circle and can be examined by non-metric smallest space analysis with only few constraints (Schlesinger and Guttman, 1969). The circular order model assumes a typical order of scale-intercorrelations, which can be examined by pairwise comparisons of scale correlations (Tracey and Schneider, 1995; Tracey, 1997). The circular stochastic process model (SPMC; Browne, 1992; Grassi et al., 2010; Nagy et al., 2019) allows for a more sophisticated investigation of correlations where equality constraints can be specified separately for the spacing between the IIP scales and their communalities (equal radius in the circle).

The SPMC can be regarded as a factor analytic model (Browne, 1992; Nagy et al., 2019), which leads to the focus of the present study, namely factor analytic approaches to the circumplex structure of the IIP. Factor models are a well-established and flexible method to describe the latent structure of personality constructs, which makes the present investigation accessible to a wide audience inside and outside of the circumplex domain. Even though factor analysis requires large samples for robust estimation of parameters and makes additional distributional assumptions, it typically has high statistical power and allows for the assessment of individual differences in underlying latent variables, making it a powerful analytic tool. Although the SPMC belongs to the class of factor models, we will primarily base our analyses on more conventional forms of factor modeling because this is in line with previous research on the IIP (Wiggins et al., 1988; Gurtman and Pincus, 2000).

Typically, the scales of the interpersonal circumplex are subsumed under the two orthogonal axes Dominance and Love, which span the circular structure (see Figure 1) (Wiggins et al., 1988; Gurtman and Pincus, 2000). However, in the case of interpersonal problems, researchers consistently find a third, general factor in addition to the two circumplex factors in both exploratory (Horowitz et al., 1988) and confirmatory analysis (Acton and Revelle, 2002; Wilson et al., 2013; Hopwood and Good, 2019; Wendt et al., 2019). Although sometimes disregarded as a response factor and eliminated by ipsatization (Horowitz et al., 1988; Alden et al., 1990), the third factor accounts for a substantial amount of shared variance between the IIP octants and seems to represent a general tendency to experience interpersonal distress (Tracey et al., 1996). Hence, the IIP octants are best represented by a three-factor solution with Distress as a general problem factor and the two circumplex factors Dominance and Love.

The three-factor model of the IIP comprising a two-factor circumplex structure for Dominance and Love and an additional third factor for Distress has been thoroughly examined by conventional confirmatory factors analysis (CFA; Wilson et al., 2013; Wendt et al., 2019). Typically, CFA is performed in order to estimate simple structure loading patterns that are based on some freely estimated large loadings and some loadings that are fixed to zero. It is, however, less clear in the context of circumplex models which exact loadings should be fixed to the values expected from the circumplex pattern. It is possible to specify a variety of CFA models with fewer or more constraints regarding the circumplex pattern. In the present study, we follow Wilson et al. (2013) by fixing all circumplex loadings to specific values, leading to the perfect circumplex model estimated by conventional, maximum-likelihood CFA (MCFA). However, model fit can only be assessed for the three-factor model as a whole when conducting conventional MCFA. Hence, the fit of the circumplex pattern for Dominance and Love cannot be disentangled from the fit of the general problem factor Distress. Loadings on Dominance and Love are fixed to the ideal circumplex and can only be assessed individually by modification indices and misspecification analysis (Saris et al., 1987, 2009). An overall assessment of the circumplex pattern is thus not possible when testing the perfect circumplex model within MCFA. Furthermore, a series of post-data model modifications may capitalize on chance and cannot be recommended without caution (MacCallum et al., 1992).

In order to overcome these problems, Bayesian CFA (BCFA; Muthén and Asparouhov, 2012) may be a convenient method for flexible specification of a higher-level model comprising a circumplex structure. The advantage of BCFA is that the loading circumplex can be specified by means of priors for the loading variance so that the large number of circumplex loadings needs not to be fixed to specific values as in conventional MCFA. Since conventional MCFA only allows for the specification of the exact ideal circumplex loadings or for completely freeing single loadings on the basis of significant modification indices, it does not allow for an overall investigation of the proximity of the loadings to the ideal circumplex. In contrast, the specification of priors for the loading variance in BCFA allows for an investigation of the overall proximity of the posterior loading pattern to the ideal loading circumplex because the estimated loadings are allowed to be different from the *a priori* specified circumplex loadings. Therefore, we will investigate the three-factor model of the IIP comprising a circumplex structure for Dominance and Love by both conventional MCFA and BCFA. Circumplex loadings might also be analyzed by means of exploratory factor analysis with subsequent target rotation (Browne, 2001). Like BCFA, target rotation also allows for a less restrictive estimation of circumplex loadings than MCFA. We therefore compare the results of target-rotated exploratory factor analysis (TEFA) with MCFA and BCFA. Whereas, exploratory analysis with subsequent target rotation toward the perfect circumplex has already been used for the investigation of circumplex models in other contexts (Wiggins et al., 1988; Jacobs and Scholl, 2005; Horowitz et al., 2017), BCFA is a rather new method that has until now rarely been used in the context of circumplex models. Therefore, and because the IIP has previously been analyzed by means of MCFA (Wilson et al., 2013; Wendt et al., 2019), the focus of the present study is on the comparison of MCFA with BCFA. However, we will also include TEFA and an analysis of the SPMC of the IIP scales to provide a comprehensive description of the circumplex model.

**Figure 2:**
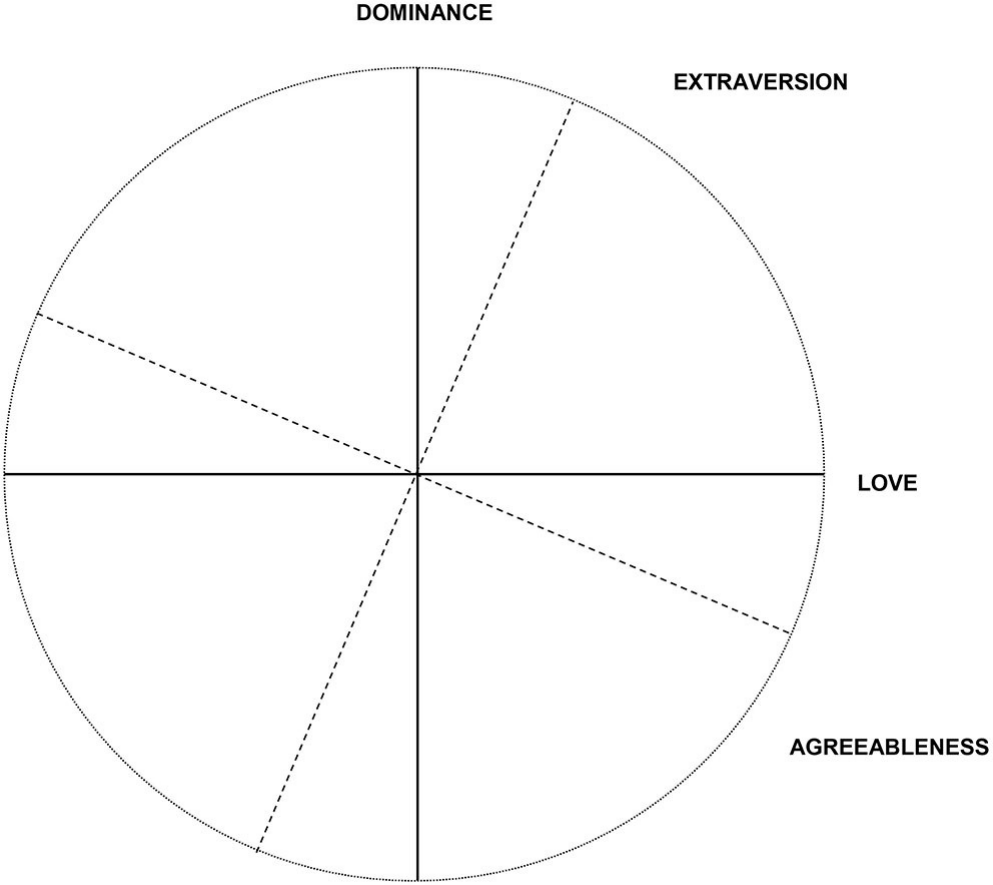
Big Five agreeableness and extraversion within the interpersonal circumplex (see also DeYoung et al., 2013, adaptation approved).

In addition to testing the internal validity of the IIP higher-level structure, we will include external criteria in the MCFA and BCFA to account for the external validity of the structural model. For this end, we will include three of the Big Five traits that are inherently linked to interpersonal behavior and possibly problems in this domain—Agreeableness, Extraversion, and Neuroticism (McCrae and Costa, 1989). Agreeableness and Extraversion have been aligned on the interpersonal circumplex in previous studies (see Figure 2; McCrae and Costa, 1989; DeYoung et al., 2013; Barford et al., 2015). According to these findings, we hypothesize that Extraversion should have positive loadings on Dominance and Love, whereby the size of the loading should be larger for Dominance than for Love. We expect Agreeableness to be positively linked to Love and negatively linked to Dominance, whereby the first loading should be larger in size than the latter. Since the IIP focuses on interpersonal problems, we hypothesize that Neuroticism may be positively associated with the IIP's general problem factor Distress.

Furthermore, we will also include subclinical grandiose narcissism in the MCFA/BCFA models as a more specific trait associated with interpersonal behavior and problems (Dickinson and Pincus, 2003; Miller et al., 2012). Based on an exploratory factor analysis of interpersonal behavior, grandiose narcissism seems to be positively associated with Dominance and negatively associated with Love (Miller et al., 2012). Regarding interpersonal problems, grandiose narcissistic personality types report domineering and vindictive interpersonal problems, however, while denying greater interpersonal distress in general. In a clinical sample, narcissism was found to be linked to greater interpersonal distress in general and a domineering, vindictive, and intrusive interpersonal style (Ogrodniczuk et al., 2009). Given that a domineering interpersonal style was associated with narcissism in all these findings, we expect subclinical grandiose narcissism to positively load on Dominance. Regarding the general problem factor Distress, we do not expect grandiose narcissism in its subclinical form to be associated with a high overall level of self-perceived interpersonal problems (Dickinson and Pincus, 2003). This is because individuals with high subclinical grandiose narcissism tend to overestimate themselves and might not perceive or report possible shortcomings in their interactional behavior. If the threshold to clinical narcissism is trespassed, however, highly narcissistic individuals might be confronted with greater obstacles in the interaction with others and hence report a greater general level of interpersonal distress (Ogrodniczuk et al., 2009).

Given the well-fitting three-factor model of the IIP scales, it has been suggested that higher-level scores should be used for Dominance, Love, and Distress (Wendt et al., 2019). Higher-level scores are important both in research (Devlieger and Rosseel, 2017; Zitzmann and Helm, 2021) and in diagnostics setting, where manifest indicators of the latent factors are needed. As there may even be theoretical shifts in the meaning of factor scores as opposed to the underlying factors (Beauducel, 2005), the validity of higher-level scores should be examined in addition to the underlying structural model. Higher-level scores of the IIP represent a parsimonious description of interpersonal problems, which nonetheless include the information from all IIP octants. However, even though a well-fitting three-factor model is a good prerequisite for using higher-level scores, it does not completely define the corresponding higher-level scores because different higher-level scores can be computed from the same CFA model. Therefore, we will investigate the reliability and validity of different higher-level scoring methods for Dominance, Love, and Distress in greater detail.

Two kinds of scores representing the higher-level structure will be examined. First, traditional latent factor score estimates (i.e., regression score estimates) from MCFA comprising higher-level factors and the intended circumplex structure are of interest. Although the specification of prior variances in BCFA allows for a flexible modeling of circumplex structure, the computation of mean plausible values as individual factor score estimates for BCFA has been discouraged because they are prone to bias (Wu, 2005; Lüdtke and Robitzsch, 2017). We therefore refrain from computing mean plausible values from BCFA in the present context.

Second, weighted sum scores are of interest because they allow for a direct specification of the circumplex structure, so that they do not require large data sets for preliminary CFA and can be easily computed in individual diagnostics settings as well. According to Wendt et al. (2019), weighted sum scores for the IIP are computed by


(1) $$\begin{array}{l} \begin{array}{ccc} Dominance & = & PA+(0.71\times NO)+(0.71\times BC)-(0.71\;\times FG)-(0.71\;\times JK)-HI\\ Love & = & LM+(0.71\times NO)+(0.71\times JK)-(0.71\;\times BC)-(0.71\;\times FG)-DE \end{array}\\ \begin{array}{ccc} \;Distress & = & \;\frac{PA\;+\;BC\;+\;DE\;+\;FG\;+\;HI\;+\;JK\;+\;LM\;+\;NO}{8} \end{array} \end{array}$$

The scores for all scales PA to NO are unit-weighted sum scores computed from the corresponding items of each scale.

After investigating the three-factor model of the IIP by MCFA, BCFA and TEFA, we will perform a thorough examination of reliability and validity of the corresponding higher-level scores using the two scoring methods mentioned above. For all analyses, we will use loadings and reproduced correlations between the IIP octants as implied by the respective scoring method. For internal validity, we will investigate congruences between the scoring-implied loadings and factor loadings from the three-factor model (Lorenzo-Seva and ten Berge, 2006). Since the IIP is a circumplex-based measure, we will also analyze the circumplexity of scoring-reproduced correlations between the IIP octants by the SPMC (Browne, 1992; Grassi et al., 2010).

Lastly, we will examine the correlational pattern of the different scores with the external criteria that are also investigated for the structural model and expect a similar pattern for the higher-level scores as hypothesized for the factors. We expect Agreeableness scores to be positively correlated with Love scores and negatively correlated with Dominance scores, whereby the first correlation should be larger in size than the latter. As for the Distress factor, we hypothesize that Neuroticism scores should be positively correlated with Distress scores. Regarding subclinical grandiose narcissism, we expect it to be positively correlated with Dominance scores. However, we do not expect narcissism scores in their subclinical form to be correlated with Distress scores as indicators of an overall level of interpersonal problems.

## Materials and Methods

### Participants and Procedure

We conducted an online-questionnaire study with a battery of inventories that were used for two distinct projects. Some of the data were also used for illustration purposes within a statistics class of the Master of Science program in Psychology at the University of Bonn. The study was approved by the institutional ethics board of the University of Bonn and conducted in line with the Declaration of Helsinki. Participants were recruited by staff members and students from the Institute of Psychology. All participants volunteered to take part in the study, which took ~45–60 min. Participants received course credit for participation if needed for their degree. A total of 999 participants took part in the study. For the final data, we included only those participants who filled out all items of the relevant measures of this study. The final dataset consisted of 822 participants (516 female, 306 male), who were 16 to 89 years old (*M* = 32.68, *SD* = 14.79).

### Measures

#### Interpersonal Problems

We used the German version of the IIP (Horowitz et al., 2017), a 64-item measure with eight scales representing the circumplex octants of interpersonal problems [LM/Overly nurturant (0°), NO/Intrusive (45°), PA/Domineering (90°), BC/Vindictive (135°), DE/Cold (180°), FG/Socially avoidant (225°), HI/Nonassertive (270°), and JK/Exploitable (315°)] and eight items per scale. For each item, participants rated on a 5-point Likert scale the extent to which they agreed to the statement (*not at all* to *very much*). Items included statements on having difficulties in certain interpersonal behaviors and statements on excessive display of certain behaviors. For example, participants rated items like “It is hard for me to trust other people” (BC/Vindictive) and “I try to please other people too much” (LM/Overly nurturant). Scores for the eight scales were computed by adding all items for each scale, resulting in eight unit weighted sum scores. Cronbach's alpha of the IIP scales ranged from α = 0.71 (NO/Intrusive) to α = 0.85 (HI/Nonassertive).

#### Big Five

We measured Extraversion, Agreeableness, and Neuroticism from the Big Five by a 40-item questionnaire based on the International Personality Item Pool (IPIP40; Hartig et al., 2003). The IPIP40 has been developed specifically for online-testing purposes (Goldberg, 1999). It has good psychometric properties and shows convergent validity with the well-established NEO-FFI (Borkenau and Ostendorf, 1993). It measures each of the Big Five scales by eight statements about an individual's typical tendencies, some of which are phrased in an inverted manner. Items include statements like “I feel good the way I am” (Neuroticism, -), “I make friends easily” (Extraversion), and “I respect others” (Agreeableness). Participants rated the extent to which the given statement applied to them on a 5-point Likert scale ranging from 1 (*very little accurate)* to 5 *(very accurate)*. Cronbach's alpha was α = 0.83 (Extraversion), α = 0.70 (Agreeableness), and α = 0.88 (Neuroticism) for the relevant IPIP40 scales.

#### Grandiose Narcissism

To measure subclinical grandiose narcissism, our participants filled out a short version of the German Narcissistic Personality Inventory (NPI-17, von Collani, 2014). It is an economic one-scale measure with 17 items stating grandiose narcissistic tendencies, which participants rate on a 5-point Likert scale from 1 (*very little accurate)* to 5 *(very accurate)*. Items include statements like “I am more competent than other people” and “I am an extraordinary person.” Cronbach's alpha for the NPI-17 was α = 0.89.

## Results

We used R version 4.1.0 for MCFA and SPMC analysis, Mplus version 8.6 for BCFA and TEFA, and SPSS version 27 for correlations and additional analyses. R lavaan was used for MCFA because it allows for a more detailed misspecification analysis of MCFA loadings than Mplus.

Before investigating the higher-level model of the IIP by MCFA and BCFA, we analyzed the correlational pattern of the IIP octants as implied by the SPMC (Browne, 1992; Nagy et al., 2019). We used CircE (Grassi et al., 2010), an R implementation of Browne's CIRCUM program, to evaluate the SPMC of the 8 x 8 correlation matrix of the IIP scales. As can be seen in Table 1, we used three combinations of equality constraints on communalities of the IIP scales and spacing between them. Equal spacing corresponded to constant 45° displacements in the circle, and equal communalities corresponded to an equal radius for all octants. For the original octant scales, the most parsimonious equal-spacing/equal-communalities model did not yield good fit (see Table 1). However, freeing the angular position (spacing) of the scales greatly improved model fit. CircE yielded that the minimum score correlation found for IIP scales that were at a 180° distance from each other was *r* = 0.001. As this was the minimum correlation, it indicated that the IIP scales showed an overall positive correlation with each other, supporting the importance of a third, general factor causing the positive correlations in addition to the two circumplex factors.

**Table 1:** CircE results of the inventory of interpersonal problems for original and reproduced correlations of the octant scales.

**Correlations from**	**Constraints**		**Goodness-of-fit measures**			
	**Spacing**	**Communalities**	***CFI***	***GFI***	***SRMR***	***RMSEA***
Original octants	Equal	Equal	0.91	0.91	0.14	0.12
	Unequal	Equal	0.96	0.96	0.06	0.10
	Unequal	Unequal	0.98	0.97	0.04	0.10
MCFA regression scores	Equal	Equal	0.94	0.91	0.10	0.13
	Unequal	Equal	0.97	0.95	0.06	0.11
	Unequal	Unequal	0.99	0.99	0.05	0.07
Weighted sum scores	Equal	Equal	0.95	0.93	0.10	0.11
	Unequal	Equal	0.99	0.99	0.05	0.06
	Unequal	Unequal	0.99	0.99	0.05	0.06

*CircE tests the Circular Stochastic Process Model (Browne, 1992). Equal spacing refers to constant 45° displacements of the octants. Equal communalities refer to a constant radius in the circle. MCFA scores were regression factor scores. MCFA, Confirmatory factor analysis with maximum-likelihood estimation; CFI, Comparative fit index; GFI, Goodness-of-fit index; SRMR, Standardized root mean square residual; RMSEA, Root mean square error of approximation*.

### Validity of the Three-Factor Model by MCFA and BCFA

We followed Wilson et al. (2013) and used a bi-factor model, comprising three orthogonal factors: Distress as a general factor for interpersonal problems and Dominance and Love as the two circumplex factors. For MCFA, we used maximum likelihood estimation with robust standard errors to counteract effects of violated multivariate normality. We z-standardized all IIP scales before entering them into the analysis to level out possible differences in circumplex loadings due to different variances of the IIP scales. In MCFA, loadings on Dominance and Love were fixed according to the circumplex model (Table 2). The same loadings were entered as the mean of normally distributed priors in BCFA with a prior variance of σ^2^ = 0.01. It has been recommended to start with smaller prior variances and to increase the prior variance in a second step (Asparouhov et al., 2015). We therefore performed the second BCFA with a prior variance of σ^2^ = 0.1. In both analyses, the loadings of PA on Dominance and of LM on Love were fixed to 1 for scaling adjustments in BCFA. In both MCFA and BCFA, loadings for Distress were estimated freely, whereby the variance of Distress was fixed to σ^2^ = 1 to obtain an identified model. All factors were set to be uncorrelated. Zitzmann and Hecht (2019) noted that the precision of the estimation should be controlled for by means of the potential scale reduction (PSR). We therefore provide the trace plot of the parameter with the greatest PSR value (see Supplementary Figure S1). For both BCFA models, the maximum PSR converged to 1.001 indicating a high precision of the estimation.

**Table 2:** Loadings from the three-factor model (MCFA and BCFA) and from higher-level scoring models of the inventory of interpersonal problems.

**Factor**		**Factor model**		**Higher-level scoring model**	
	**Scale**	**MCFA**	**BCFA**	**MCFA scores**	**Weighted sums**
Dominance(circumplex)	PA	1	1	0.71	0.70
	BC	0.71	0.62^***^	0.41	0.38
	DE	0	0.06	−0.01	−0.03
	FG	−0.71	−0.54^***^	−0.40	−0.41
	HI	-1	−0.91^***^	−0.60	−0.62
	JK	−0.71	−0.76^***^	−0.40	−0.44
	LM	0	−0.28^***^	−0.03	−0.11
	NO	0.71	0.64^***^	0.55	0.54
Love(circumplex)	PA	0	−0.24^***^	−0.06	−0.09
	BC	−0.71	−0.80^***^	−0.45	−0.47
	DE	-1	−0.88^***^	−0.57	−0.60
	FG	−0.71	−0.71^***^	−0.51	−0.48
	HI	0	0.19^***^	0.01	0.04
	JK	0.71	0.83^***^	0.45	0.42
	LM	1	1	0.61	0.58
	NO	0.71	0.83^***^	0.58	0.60
Distress(general)	PA	0.66^***^	0.52^***^	0.69	0.69
	BC	0.49^***^	0.61^***^	0.53	0.64
	DE	0.47^***^	0.65^***^	0.52	0.60
	FG	0.59^***^	0.74^***^	0.63	0.64
	HI	0.64^***^	0.62^***^	0.67	0.64
	JK	0.73^***^	0.62^***^	0.75	0.67
	LM	0.64^***^	0.64^***^	0.65	0.59
	NO	0.65^***^	0.50^***^	0.66	0.61

*Dominance, Love, and Distress were uncorrelated in MCFA and BCFA (bi-factor model). In MCFA, loadings were fixed according to the circumplex model and inserted as priors for BCFA. For scaling adjustments, the loadings of PA on Dominance and of LM on Love were fixed to 1 in BCFA. MCFA scores were regression factor scores. MCFA, Confirmatory factor analysis with maximum likelihood estimation; BCFA, Bayesian confirmatory factor analysis*.

*** *p < 0.001 (two-tailed)*.

Model fit of the bi-factor circumplex model by MCFA was χ^2^(18) = 207.13, *p* < 0.001, *CFI* = 0.94, *SRMR* = 0.09, *RMSEA* = 0.11, 90% CI [0.10, 0.13]. Model fit of the bi-factor circumplex BCFA model based on a prior variance of σ^2^ = 0.01 was 95% CI [34.10, 86.48] for χ^2^(40), the χ^2^-based posterior predictive *p-*value was *p* < 0.001, *CFI* = 0.98, *RMSEA* = 0.09, 90% CI [0.08, 0.10]. For a prior variance of σ^2^ = 0.1, model fit was 95% CI [29.59, 80.05] for χ^2^(40), the χ^2^-based posterior predictive *p-*value was *p* < 0.001, *CFI* = 0.98, *RMSEA* = 0.09, 90% CI [0.08, 0.11]. Posterior predictive *p-*values were the likelihood-ratio χ^2^-statistic as the Mplus default. Since the fit of the two BCFA models was rather similar, we also computed Tucker's congruence coefficient *c* (Lorenzo-Seva and ten Berge, 2006) of the loadings on Dominance and Love with the ideal circumplex loadings for both BCFA models. For a prior variance of σ^2^ = 0.01, congruences were *c* = 0.984 for Dominance and *c* = 0.984 for Love. For a prior variance of σ^2^ = 0.1, congruences were *c* = 0.976 for Dominance and *c* = 0.977 for Love. As one would expect, the congruence with the ideal circumplex was a bit higher for the BCFA model based on a prior variance of σ^2^ = 0.01. We therefore used this model for further analyses.

We saved MCFA scores estimated from the parameters of the MCFA model. By Mplus default, these scores are regression scores (McDonald, 2011), that is, these scores have the maximal correlation of a linear combination of the measured variables with the corresponding factor. The weights that were multiplied with the IIP scales' scores to obtain MCFA regression scores for the present dataset can be found in the Supplement (Supplementary Table S1).

Maximum likelihood based TEFA resulted in an acceptable model fit χ^2^(5) = 62.03, *p* < 0.001, *CFI* = 0.99, *SRMR* = 0.01, *RMSEA* = 0.12, 90% CI [0.09, 0.15]. The ideal circumplex loadings of Dominance and Love were used as target loadings for orthogonal target rotation. No target rotation was performed for the Distress factor. The congruence of the TEFA loadings with the ideal circumplex loadings was *c* = 0.798 for Dominance and *c* = 0.735 for Love. When congruence scores were adjusted for differences in communalities (Kaiser normalization), they were *c* = 0.812 for Dominance and *c* = 0.714 for Love. As congruences were substantially smaller for TEFA than for BCFA, we did not conduct follow-up TEFA analyses or compute TEFA factor scores.

Table 2 shows the ideal circumplex loadings as specified in MCFA as well as the BCFA loadings, so that one can see their deviations from perfect circumplexity. In BCFA, deviations from the perfect circumplex were largest (|>0.20|) for two loadings that were fixed to 0 in MCFA: The loading of the LM/Overly nurturant scale on Dominance and the loading of the PA/Domineering scale on Love, hence, for scales that were supposed to each be perfectly aligned on the opposite factor (see Figure 1). We also conducted an additional misspecification analysis of MCFA circumplex loadings (Saris et al., 1987, 2009), which indicated large expected parameter changes for the two respective MCFA loadings in the direction of the BCFA loadings. Loadings on the general factor Distress were positive and significant with *p* < 0.001 for all IIP octants for both MCFA and BCFA. Congruence between the corresponding MCFA/BCFA Distress factors was *c* = 0.999.

Additionally, we investigated shifts in model fit for MCFA and BCFA when error terms were allowed to be correlated. We selected those correlations with a modification index >10 in the first MCFA to be freed in a follow-up MCFA, resulting in 12 correlated error terms, seven of which were significant at *p* < 0.05 (MCFA model fit: χ^2^(6) = 92.70, *p* < 0.001, *CFI* = 0.97, *SRMR* = 0.08, *RMSEA* = 0.14, 90% CI [0.12, 0.17]). These 12 error terms were freely estimated in BCFA with a mean of zero and a prior variance of σ^2^ = 0.01 (BCFA model fit: 95% CI [−23.86, 29.86] for χ^2^(52), χ^2^-based posterior predictive *p* = 0.412, *CFI* > 0.99, *RMSEA* = 0.04, 90% CI [0.00, 0.12]). Only two correlated error terms were significant in BCFA, indicating that the increased flexibility in the estimation of circumplex loadings tends to reduce the size of correlated errors.

As BCFA resulted in fewer significant error correlations than MCFA, the enhanced flexibility of BCFA estimation could also result in a more robust estimation of model fit. This was investigated by splitting the sample in two equally sized subsamples. The models were specified as before but without correlated error terms. In addition to the previously reported fit indices, we also report the Bayesian Information Criterion (*BIC*) to assess the variation of model fit (see Supplementary Table S2). The difference in model fit between the MCFA models in the two subsamples was slightly larger for the *CFI, RMSEA*, and *BIC* than for the BCFA models, indicating that BCFA model fit was less affected by variations in the two samples.

**Figure 3:**
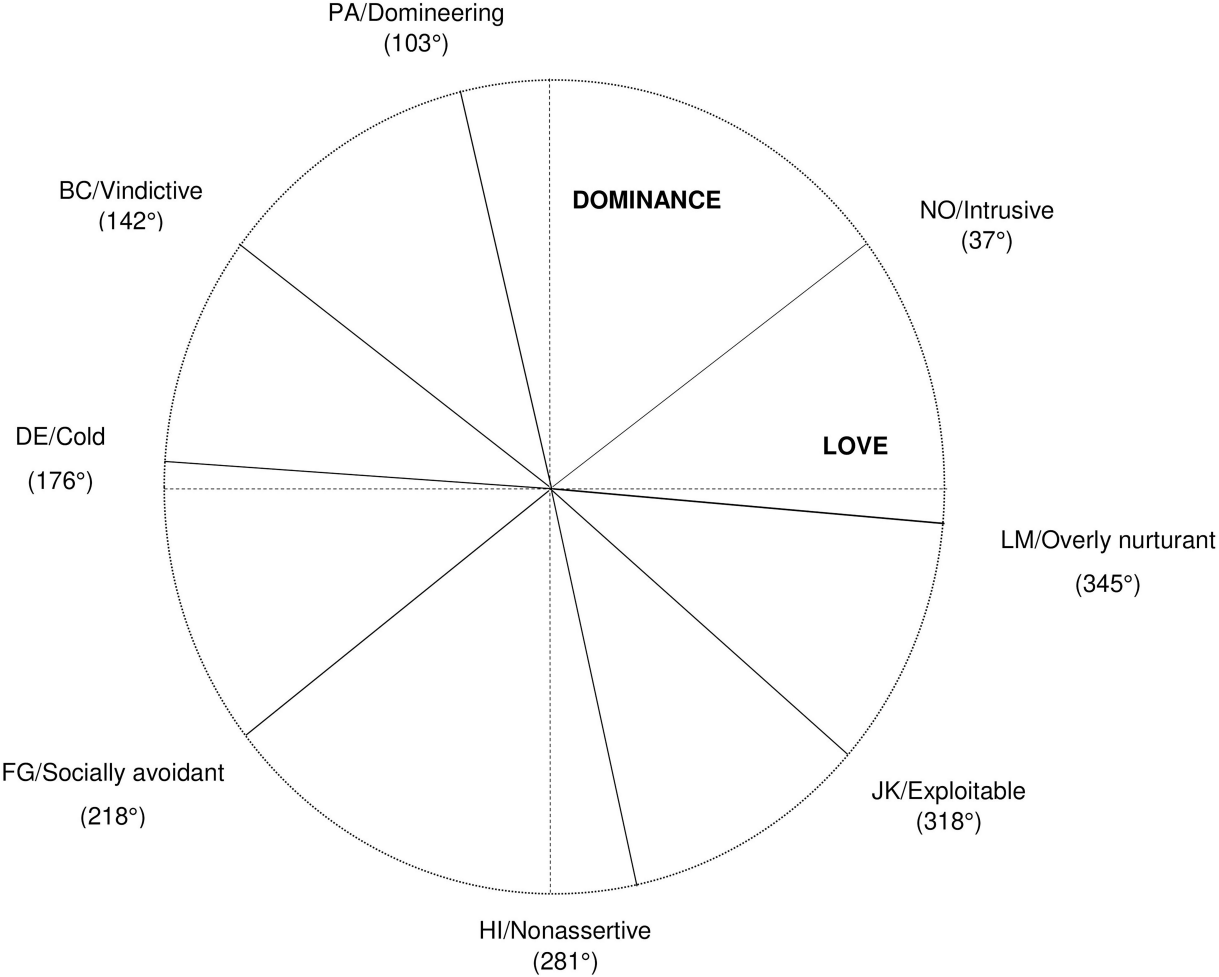
Circumplex structure of the inventory of interpersonal problems modeled by Bayesian confirmatory factor analysis. Circumplex angles with relation to the Dominance and Love factor were obtained by applying trigonometric functions to posterior loadings from Bayesian confirmatory factor analysis. The radius of the scales was kept at one by Kaiser-normalizing all loadings prior to trigonometric transformation. The dashed lines represent the Dominance and Love factor, the solid lines represent the scales from the inventory of interpersonal problems.

All further analysis (external validity of the IIP's structural model and the computation of factor scores) was based on the orthogonal three-factor model without correlated errors. In this model, loadings on Dominance and Love were fixed to the perfect circumplex in MCFA and entered as priors in BCFA with a prior variance of σ^2^ = 0.01 (see Table 2). The number of iterations in BCFA was adjusted to result in PSR ≤ 1.001. The circumplex pattern of the BCFA posterior loadings can be inspected in Figure 3.

In addition to the analysis of internal validity, we embedded external criteria in MCFA and BCFA to support the external validity of the IIP's structural model (Table 3). External measures were entered in the CFA as z-standardized sum scores like the IIP scales. We included Extraversion, Agreeableness, and Neuroticism scores from the IPIP40 (Hartig et al., 2003) and NPI-17 scores measuring subclinical narcissism (von Collani, 2014). Model fit was similar to model fit without the external measures. Model fit for the MCFA model with the Big Five measures was χ^2^(36) = 335.00, *p* < 0.001, *CFI* = 0.94, *SRMR* = 0.09, *RMSEA* = 0.10, 90% CI [0.09, 0.11]. MCFA model fit for the inclusion of subclinical grandiose narcissism was χ^2^(23) = 248.41, *p* < 0.001, *CFI* = 0.94, *SRMR* = 0.09, *RMSEA* = 0.11, 90% CI [0.10, 0.12]. Model fit for the BCFA model was 95% CI [113.73, 179.30] for χ^2^(55), χ^2^-based posterior predictive *p* < 0.001, *CFI* = 0.97, *RMSEA* = 0.08, 90% CI [0.08, 0.09] with Extraversion, Agreeableness, and Neuroticism. The BCFA model with grandiose narcissism resulted in a model fit of 95% CI [67.24, 124.26] for χ^2^(45), χ^2^-based posterior predictive *p* < 0.001, *CFI* = 0.98, *RMSEA* = 0.09, 90% CI [0.08, 0.10].

**Table 3:** Confirmatory factor analysis of the inventory of interpersonal problems with external measures.

**Scale/** **variable**	**MCFA**			**BCFA**		
	**Dominance**	**Love**	**Distress**	**Dominance**	**Love**	**Distress**
PA	1	0	0.48^***^	1	−0.26^***^	0.51^***^
BC	0.71	−0.71	0.59^***^	0.64^***^	−0.79^***^	0.61^***^
DE	0	-1	0.63^***^	0.07	−0.85^***^	0.64^***^
FG	−0.71	−0.71	0.74^***^	−0.57^***^	−0.74^***^	0.75^***^
HI	-1	0	0.63^***^	−0.92^***^	0.21^***^	0.62^***^
JK	−0.71	0.71	0.63^***^	−0.76^***^	0.84^***^	0.61^***^
LM	0	1	0.66^***^	−0.29^***^	1	0.64^***^
NO	0.71	0.71	0.50^***^	0.69^***^	0.86^***^	0.51^***^
Extraversion	1.00^***^	0.81^***^	−0.36^***^	0.87^***^	0.77^***^	−0.35^***^
Agreeableness	–0.96^***^	0.58^***^	−0.20^***^	–0.94^***^	0.68^***^	−0.23^***^
Neuroticism	−0.14^*^	−0.19^**^	0.60^***^	−0.14^*^	−0.16^**^	0.61^***^
PA	1	0	0.66^***^	1	−0.24^***^	0.52^***^
BC	0.71	−0.71	0.49^***^	0.61^***^	−0.80^***^	0.61^***^
DE	0	-1	0.48^***^	0.06	−0.89^***^	0.65^***^
FG	−0.71	−0.71	0.59^***^	−0.53^***^	−0.70^***^	0.73^***^
HI	-1	0	0.64^***^	−0.94^***^	0.18^***^	0.62^***^
JK	−0.71	0.71	0.73^***^	−0.75^***^	0.83^***^	0.62^***^
LM	0	1	0.68^***^	−0.27^***^	1	0.64^***^
NO	0.71	0.71	0.65^***^	0.63^***^	0.83^***^	0.50^***^
Grandiose narcissism	1.05^***^	0.04	0.01	0.94^***^	−0.05	0.04

*Boldface entries represent results for which hypotheses were formulated. Dominance, Love, and Distress were uncorrelated in MCFA and BCFA (bi-factor model). In MCFA, loadings were fixed according to the circumplex model and inserted as priors for BCFA. For scaling adjustments, the loadings of PA on Dominance and of LM on Love were fixed to one in BCFA. MCFA scores were regression factor scores. MCFA, Confirmatory factor analysis with maximum likelihood estimation; BCFA, Bayesian confirmatory factor analysis*.

* *p < 0.05 (two-tailed)*.

** *p < 0.01 (two-tailed)*.

*** *p < 0.001 (two-tailed)*.

Results followed a similar pattern for MCFA and BCFA (Table 3) and will be reported for those loadings that are most relevant within the theoretical framework of the IIP (see Introduction). Extraversion loaded positively on Dominance and Love, whereby the loading on Dominance was greater than the loading on Love. Agreeableness had a negative loading on Dominance and a positive loading on Love, however, the latter to a smaller degree. Neuroticism showed large negative loadings on Distress as an overall measure of interpersonal problems. Lastly, subclinical narcissism showed large and positive loadings on Dominance and zero loadings on Love and Distress.

### Reliability and Validity of IIP Higher-Level Scores

We analyzed higher-level scores for Dominance, Love, and Distress using regression factor scores from MCFA and weighted sum scores. Weighted sum scores were computed according to Equation 1. The weights to obtain MCFA regression scores from the IIP scales' scores can be seen in the (Supplementary Table S1). The MCFA regression scores were perfectly predicted by the IIP octant scales (*R*^2^ = 1). As can be seen in Table 4, correlations between corresponding IIP higher-level scores from the two scoring methods were very large with *r*(820) > 0.99, *p* < 0.001, for all scores Dominance, Love, and Distress. With regard to intercorrelations of higher-level scores within each scoring method, the largest absolute correlation was found between the MCFA regression scores for Love and Dominance (see Table 4). The two circumplex scores Love and Dominance were uncorrelated with Distress for MCFA regression scores, whereas the weighted sum score for Dominance showed a slight negative correlation with Distress.

**Table 4:** Correlations between higher-level scores of the inventory of interpersonal problems and reliability estimates.

	**RDOM**	**RLOV**	**RGEN**	**SDOM**	**SLOV**	**SGEN**
RDOM	**0.90**	–0.31^***^	–**0.03**	0.99^***^	−0.27^***^	0.01
RLOV		**0.91**	**<0.01**	−0.28^***^	0.99^***^	0.00
RGEN			**0.95**	−0.15^***^	−0.04	>0.99^***^
SDOM				**0.91**	–0.24^***^	–0.11^**^
SLOV					**0.91**	–**0.04**
SGEN						**0.95**

*Reliability estimates of the higher-level scores are displayed in the main diagonal. Reliability estimates, correlations between corresponding scores from different scoring methods, and intercorrelations between scores of the same scoring method are written in boldface letters. DOM, Dominance; LOV, Love; GEN, General. Preceding letters: R, Regression factor scores from confirmatory factor analysis with maximum likelihood estimation; S, Weighted sum scores*.

** *p < 0.01 (two-tailed)*.

*** *p < 0.001 (two-tailed)*.

For all following analyses of reliability and validity of IIP higher-level scores, we used the scoring model implied by the two scoring methods. We computed regression-component loadings of the scoring models (Schönemann and Steiger, 1976; Beauducel, 2005) as


(2) $$\Lambda_{\;}=\Lambda^{*}C^{-1}\;,$$

where Λwere the standardized loadings of the IIP octants on the higher-level scores, Λ^*^ were the Pearson product-moment correlations between the octants and higher-level scores, and *C* were the intercorrelations between the higher-level scores. The corresponding reproduced correlation matrix between the IIP octants Σ_*r*_ was computed by


(3) $$\Sigma_{r}\;=\;\Lambda_{\;}C\;{\Lambda^{\prime }}_{\;}.\;$$

The coefficients of congruence of the regression component loadings with the factor loadings of the models were very high (*c* ≥ 0.99) for all types of scores and factor models, including congruences between higher-level scores with BCFA factor loadings.

We computed reliability estimates for the higher-level scores by the following formula, originally designed for two sets of parallel observed variables (Cliff, 1988; p. 277, Eq. 4):


(4) $$R_{tts}=diag(diag{\left(B^{\prime }\Sigma_{11}B\right)}^{-\frac{1}{2}}\;B^{\prime }\Sigma_{12}B\;diag{\left(B^{\prime }\Sigma_{22}B\right)}^{-\frac{1}{2}}-),\;$$

where *B* contained the weights obtained from a multiple linear regression in which the higher-level scores were predicted by the IIP octant scales (see Supplementary Table S1). Originally, Σ_11_ should represent the intercorrelations of the first variable set, Σ_22_ of the second variable set, and Σ_12_ the correlations between the first and the second variable set. We assumed that Σ_11_ = Σ_22_ (Beauducel et al., 2016) and used intercorrelations of the octant scales as estimators for Σ_11_ as well as Σ_22_ and the intercorrelations of the octants reproduced by the scoring model, Σ_*r*_, as estimators for Σ_12_. Reliability estimates were very high and very similar for the two scoring methods (Table 4). They were largest for the general problem factor Distress with *r*_*tts*_ = 0.95, followed by Love (*r*_*tts*_ = 0.91), and lastly Dominance with *r*_*tts*_ = 0.90 for MCFA regression scores and *r*_*tts*_ = 0.91 for weighted sum scores.

We analyzed the circumplexity of the reproduced correlations Σ_*r*_ implied by the two higher-level scoring models by the SPMC and compared them to the results for the original octant scales (Table 1). The best approximation to perfect circumplexity was found for reproduced correlations from the weighted sum scores for Dominance, Love, and Distress for all combinations of equality constraints on spacing and communalities. MCFA regression scores improved model fit as compared to the original octant scales for some, but not all SPMC conditions and fit indices.

For the unequal-spacing/equal-communalities SPMC model, we also inspected the estimated angles for the different correlation matrices (Table 5) in addition to overall model fit. The angle of the LM/Overly nurturant scale was set to zero, from which the other angles were estimated. We quantified the proximity to the ideal circumplex of the estimated angles by the gap difference test (GDIFF; Upton and Fingleton, 1989), with smaller values indicating greater circumplexity of the scores. The GDIFF test comprises all squared differences between ideal and observed angles. We computed GDIFF with degrees rather than radians and used its square root to facilitate interpretation of results as


(5) $$\sqrt{GDIFF}=\;\sqrt{\frac{1}{8}\sum_{i\;=\;1}^{8}{\left(\theta_{i}-\;\theta_{id}\right)}^{2}}\;,$$

**Table 5:** Circular angles of the inventory of interpersonal problems for original and reproduced correlations.

**Scale**	**Ideal circumplex**	**Original octants**	**MCFA scores**	**Weighted sums**
LM	0°	0°	0°	0°
NO	45°	63°	55°	57°
PA	90°	137°	122°	128°
BC	135°	176°	163°	169°
DE	180°	208°	196°	202°
FG	225°	244°	232°	238°
HI	270°	303°	289°	296°
JK	315°	335°	329°	331°

*Original octants are the eight original IIP scales. Angles were estimated with CircE and equal communalities of the octants. The angle for LM was fixed to 0° for all analyses. MCFA scores were regression factor scores. MCFA, Confirmatory factor analysis with maximum-likelihood estimation*.

where θ_*i*_ was the observed angle of each of the eight IIP scales and θ_*id*_ was the ideal circumplex angle for the respective scale. Values for $\sqrt{GDIFF}$ representing overall deviation from ideal circumplex angles were greater for original than score-reproduced correlations. In descending order, we found $\sqrt{GDIFF}$ = 29.26 for original correlations, $\sqrt{GDIFF}$ = 23.26 for weighted sum scores, and $\sqrt{GDIFF}$ = 18.61 for MCFA regression scores. As can be seen in Table 5, the variation of $\sqrt{GDIFF}$ was mainly driven by spacing between NO/Intrusive, PA/Domineering, and BC/Vindictive, which was closest to constant 45° spacing for MCFA regression scores.

Parallel to the analysis of external validity for the three-factor model of the IIP, we investigated the correlational pattern of the IIP higher-level scores with Extraversion, Agreeableness, and Neuroticism scores from the IPIP40 (Hartig et al., 2003) and NPI-17 scores (von Collani, 2014). Results were in line with the results for the structural model and followed a similar pattern for MCFA regression scores and weighted sum scores (see Table 6). Extraversion correlated positively with Dominance and Love, whereby the correlation with Dominance was greater than the correlation with Love. Agreeableness showed a negative correlation with Dominance and a positive correlation with Love, however, the latter to a smaller degree. Neuroticism showed large negative correlations with Distress. Lastly, subclinical narcissism showed large and positive correlations with Dominance, slight negative correlations with Love, and zero correlations with Distress.

**Table 6:** Correlations between higher-level scores of the inventory of interpersonal problems and external measures.

	**Extraversion**	**Agreeableness**	**Neuroticism**	**Grandiose narcissism**
RDOM	0.40^***^	–0.59^***^	−0.07	0.53^***^
RLOV	0.28^***^	0.45^***^	−0.07^*^	−0.09^*^
RGEN	−0.35^***^	−0.16^***^	0.57^***^	–**0.01**
SDOM	0.45^***^	–0.56^***^	−0.14^***^	0.53^***^
SLOV	0.31^***^	0.43^***^	−0.08^*^	−0.13^***^
SGEN	−0.32^***^	−0.19^***^	0.57^***^	**0.02**

*Boldface entries represent results for which hypotheses on the correlational pattern were formulated. DOM, Dominance; LOV, Love; GEN, General. Preceding letters: R, Regression factor scores from MCFA; S, Weighted sum scores*.

* *p < 0.01 (two-tailed)*.

** *p < 0.01 (two-tailed)*.

*** *p < 0.001 (two-tailed)*.

## Discussion

There are two key findings of the present study. First, the results support the notion that BCFA (Muthén and Asparouhov, 2012) is a convenient tool to model the circumplex structure of interpersonal problems. Second, the results provide evidence that higher-level scores are reliable and valid indicators of the IIP factors for Dominance, Love, and Distress.

### Interpretation and Relevance of Results

The flexibility of BCFA parameter estimates makes it particularly applicable in circumplex settings, where a whole pattern of exact loadings is specified. By making use of priors for the loading variance in BCFA, the specified circumplex loadings are adjusted to better represent the data. The posterior loading pattern of the two circumplex factors can then be compared to the ideal circumplex by Tucker's congruence coefficient (Lorenzo-Seva and ten Berge, 2006). This procedure of comparing observed loading patterns with the ideal circumplex is also possible within exploratory analysis (Jacobs and Scholl, 2005). However, we compared the congruences of TEFA loadings with the ideal circumplex loadings and found that they were substantially smaller than the congruences of the BCFA loadings. The present study therefore indicates that BCFA is a flexible approach that allows for a closer approximation of the circumplex model of the IIP than TEFA. Hence, BCFA combines the advantages of exploratory and confirmatory analysis. Furthermore, the procedure of comparing BCFA loadings of Dominance and Love to the ideal circumplex is especially relevant when dealing with interpersonal problems, where a third general factor is involved. CFA fit indices, which measure overall model fit, are mainly driven by loadings on Distress (see also Wilson et al., 2013), whereby overall adequacy of the hypothesized circumplex pattern cannot be disentangled from the overarching three-factor structure. Regarding the overall three-factor model of the IIP, we found that model fit was acceptable for conventional MCFA and increased when BCFA was conducted, hence, when circumplex constraints were loosened by specifying priors. This effect was mainly driven by those loadings that were fixed to zero in MCFA, indicating that zero loadings could be too restrictive in empirical circumplex settings. However, the exceedingly high congruence between posterior BCFA and MCFA loadings reveals that the factors can be considered equal (Lorenzo-Seva and ten Berge, 2006). Hence, BCFA produces loadings on Dominance and Love that fulfill nearly perfect circumplex structure whilst being flexible enough to loosen empirically unrealistic constraints and thereby improving model fit. More flexible estimation of model parameters also resulted in a smaller number of substantial correlated error terms for BCFA than MCFA and in a smaller variation of model fit indices across two subsamples. Thus, BCFA seems to be less sensitive toward correlated errors and yields more robust model fit estimates than MCFA. Considering the rather large *RMSEA* values found for both MCFA and BCFA, it has been argued that *RMSEA* tends to get biased and is susceptible to falsely reject adequate models in the case of large main loadings and circumplex structure in particular (Saris et al., 1987, 2009; Rogoza et al., 2021). For example, Gurtman and Pincus (2003) used a threshold of *RMSEA* < 0.13 when assessing circumplex models, which would indicate acceptable fit for our MCFA and BCFA model.

The results of circumplexity analysis to test the SPMC (Browne, 1992; Grassi et al., 2010) suggest that strict 45° equal spacing might not be a realistic constraint for the IIP octants. SPMC model fit improved greatly when spacing between the octants was freed. Estimated angles from the unequal-spacing/equal-communalities condition suggest that spacing between LM/Overly nurturant, NO/Intrusive, and PA/Domineering is better approximated by angles larger than 45°, while spacing between the other IIP scales could be smaller than 45°. Horowitz et al. (2017) report similar results for principal component angles of the original IIP scales in the validation study of the IIP. Theoretically, PA and LM should be perfectly aligned on Dominance and Love (Gurtman, 1993; Gurtman and Pincus, 2000) and thus be unrelated with a 90° distance. However, our results suggest that individuals who are more domineering according to the IIP scales are slightly less affectionate and vice versa. Taking a closer look at the items of the scales reveals that the wording of items from the PA/Domineering scale could be responsible for the angular deviations. For example, one item says, “I try to exert influence on others to get what I want,” which could be understood as manipulative behavior and overlap with the BC/Vindictive scale. Another one says, “I am too aggressive toward others,” which can hardly be independent from content of the LM/Overly nurturant scale. While items from the NO/Intrusive scale are worded more clearly as intrusive behavior, it seems like being caring (i.e., scoring high in the LM/Overly nurturant scale) is not as close to being overinvolved in other people's lives as one could assume.

The results from MCFA and BCFA with the inclusion of external measures were in line with the hypothesized pattern and support the validity of the structural model of the IIP. Results did not differ between MCFA and BCFA, which indicates that differences between the CFA methods are most relevant when the internal validity, that is, the validity of the circumplex pattern, is concerned. Previous research has linked Extraversion and Agreeableness to interpersonal behavior in general and not interpersonal problems in particular (McCrae and Costa, 1989; DeYoung et al., 2013; Barford et al., 2015). The results of the present study indicate that the expected pattern for Dominance and Love can be transferred to interpersonal problems if the circumplex factors are separated from the general problem factor Distress. Only the positive loadings of Agreeableness on Love were smaller in size than the negative loadings of Agreeableness on Dominance, which was expected to be the other way around. However, the exact size of loadings and correlations also depends on the reliability of the scales that are administered, and we found greater Cronbach's alpha for the Extraversion scale than for the Agreeableness scale of the IPIP40 (Hartig et al., 2003). The large loadings of Neuroticism on Distress highlight that the third factor of the IIP is not only a response factor but has relevant meaning as a general factor associated with emotional and interpersonal problems (see also Tracey et al., 1996).

Our findings on grandiose narcissism and IIP higher-level factors were in line with our hypotheses and previous findings. The positive loading on Dominance is in line with previously found patterns (Dickinson and Pincus, 2003; Miller et al., 2012). While clinical narcissism seems to be associated with an overall impairment in interpersonal behavior (Ogrodniczuk et al., 2009), our results support the findings by Dickinson and Pincus (2003) and suggest that subclinical grandiose narcissism is not linked to general interpersonal distress. This finding could also be attributed to the fact that subjects with high grandiose narcissistic tendencies present themselves more favorably–that is, with fewer interpersonal problems–than what would be experienced by their surroundings.

Regarding the higher-level scores for Dominance, Love, and Distress, our results suggest that both regression scores from MCFA and weighted sum scores measure interpersonal problems with excellent reliability and good validity. Reliability estimates for Dominance, Love, and Distress scores even exceeded those of the original IIP octants, which strongly supports the use of higher-level scores. We did not include mean plausible values from BCFA into the analysis because of their susceptibility to bias in individual scoring settings (Lüdtke and Robitzsch, 2017). However, the excellent congruences of the MCFA regression scores and weighted sum scores with BCFA factor loadings indicate that they are a good representation of the IIP's higher-level structure and a feasible alternative to BCFA mean plausible values.

The results of circumplexity analysis by the SPMC (Browne, 1992; Grassi et al., 2010) for reproduced correlations from the higher-level scores indicate that correlations between the IIP octants fulfill circumplexity to a greater degree when they are reproduced from higher-level scores as opposed to the original IIP correlations. Although this is not surprising given the circumplex specifications of the underlying models, the results support the validity of IIP higher-level scores for their use in research and diagnostics. Across all SPMC conditions, weighted sum scores showed best circumplex validity. This can be attributed to the fact that weighted sum scores are based on fixed circumplex weights without any data-driven adjustments. However, all higher-level scores seem to be well-fit with respect to circumplex properties of reproduced correlations. The estimated angles suggest that higher-level scores improve circumplex spacing of the IIP octants, whereby MCFA regression scores seem to best approximate ideal 45° spacing.

Correlations between higher-level scores and external measures were in line with the hypothesized pattern and support the validity of Dominance, Love, and Distress scores for MCFA regression scores and weighted sum scores. The similarity to the findings for the factor model supports the notion that the IIP higher-level scores are a good representation of the underlying factors, also with regard to the relationship with external criteria. The findings for the correlations only differed slightly from the MCFA/BCFA findings for subclinical grandiose narcissism. While only the positive loading on Dominance was significant in the MCFA and BCFA, we also found slight positive correlations of NPI-17 scores with Love scores. These correlations suggest that subclinical grandiose narcissism could be positioned at 90–135° within the interpersonal circumplex. This hypothesis is in line with results by Nagy et al. (2019), who found the admiration aspect of narcissism to be positioned within this angular range when modeling the interpersonal circumplex by the SPMC.

Taken together, our results reveal convergence of different modeling and scoring procedures for the IIP. SPMC analysis, MCFA, BCFA, and TEFA reveal the circumplex structure in a similar fashion. Moreover, we found high convergence for the higher-level scores computed by different methods. Therefore, our results demonstrate that methodological pluralism, as it has been advocated by Zitzmann and Loreth (2021), does not preclude convergence of results. Although the results reveal that the IIP circumplex can be modeled by means of MCFA, BCFA, TEFA, and SPMC, there were nevertheless relevant differences. As in MCFA the ideal loadings are directly fixed, there is no information on the departure of single items from the circumplex unless modification indices are investigated. In contrast, BCFA, TEFA, and SPMC allow for a description of the conformity of each measured variable to the circumplex. Whereas, the circumplexity does not affect model fit of TEFA, it affects the model fit of MCFA, BCFA, and SPMC. Accordingly, MCFA, BCFA, TEFA, and SPMC must not necessarily provide converging results. Therefore, the convergence of the methods found in the present study corroborates the circumplex model of the IIP. Regarding the comparison of MCFA and BCFA, we note that MCFA resulted in more significant correlated error terms and in a more substantial variation of model fit across subsamples than BCFA. Thus, we found some advantages in using prior variances in BCFA in order to avoid over-specification of circumplex loadings. However, it follows from the perspective of Zitzmann and Loreth (2021) that researchers using BCFA for circumplex modeling should tolerate those who use another approach and should be aware of the advantages and disadvantages of their methodological choices.

### Limitations and Future Research

There are three main limitations of the present study. First, we used rather short and less differentiated scales for the external measures, which could affect the reliability of results. Future research on the topic could use measures like the Revised NEO Personality Inventory (Costa and McCrae, 2008) for the Big Five as well as more exhaustive scales for narcissism. The items of the NPI-17 only measure grandiose narcissism, that is, a tendency to overemphasize one's strengths and power whilst downplaying one's weaknesses (von Collani, 2014; Foster et al., 2015). Future research could include vulnerable narcissism, which is likely to produce different results. For example, we would expect positive correlations of vulnerable narcissism with general interpersonal distress because vulnerable narcissists are more centered around their own suffering than grandiose narcissists (Dickinson and Pincus, 2003).

The second limitation concerns the sample of our study. We did not use a random sample for the present investigation, which complicates interpretation of results for the general population, especially regarding the exact size of effects and parameters. However, our results are in line with theoretical expectations concerning the circumplex structure of the IIP, which supports the validity of the present findings. Furthermore, results remained stable when the data set was split and when different methodological approaches were applied. Moreover, we used a non-clinical sample. While research on non-clinical samples is important to set a baseline for the majority of the population, the IIP as a measure of problematic behavior is also designed for clinical use (Horowitz et al., 2017). Therefore, it should be investigated whether the results can be replicated in a clinical sample. Furthermore, one could examine whether IIP higher-level scores can differentiate between patient groups or predict treatment outcome, as previously shown for the corresponding octants (Alden and Phillips, 1990; Pincus and Wiggins, 1990; Ruiz et al., 2004; Tilden et al., 2010). The IIP higher-level scores could be particularly interesting when personality disorders are investigated because personality disorders are greatly concerned with impairments in the interpersonal domain (Pincus and Wiggins, 1990). Thereby, it would be important to include clinical interviews and ratings rather than relying solely on self-report measures because individuals with personality disorders might reach and report different conclusions about themselves than an external judge. In a clinical setting, it could also be examined which of the three higher-level scores Dominance, Love, and Distress has the greatest predictive validity for which psychological disorder and compare factor score estimates and weighted sum scores as well.

Thirdly, we used only a subset of possible models for the IIP higher-level and circumplex structure. The factor models in this study ranged from strictly constrained to the perfect circumplex in MCFA, partially constrained by priors in BCFA, to completely free and only matched to the circumplex by target rotation in TEFA. However, it would be possible to specify various factor models with different levels of constraints for both MCFA and BCFA. We also applied the SPMC to our data, which can be viewed as a specific type of CFA model that can be estimated by structural equation modeling (Browne, 1992; Nagy et al., 2019). Variants of the SPMC could be examined in greater detail for the IIP in future research. The SPMC is a very flexible approach for circumplex structure and it is applicable to a variety of structural models. Typically, the SPMC is analyzed by using maximum-likelihood estimation, but it would be possible to analyze it by Bayesian estimation with the appropriate constraints as well (see Lenk et al., 2006). Moreover, Nagy et al. (2019) provide an extended version of the SPMC, which makes it possible to include external variables to investigate the external validity of the circumplex structure (see also Etzel et al., 2021). For example, it would be interesting to enter different measures of narcissism and test their angular position within the circumplex pattern of the IIP using the SPMC.

### Conclusion and Recommendations

In summary, the present study supports the three-factor model of the IIP and the use of Bayesian modeling (Muthén and Asparouhov, 2012) in circumplex settings. The possibility to specify exact loading hypotheses whilst simultaneously allowing for enough flexibility to loosen unrealistic constraints (e.g., strict zero loadings) makes it particularly suitable for scales that comprise a circumplex structure. We also encourage researchers and practitioners to use the IIP higher-level scores Love, Dominance, and Distress. They are reliable, valid, and economic measures of interpersonal problems. We can recommend both MCFA regression scores and weighted sum scores. Which of these options should be preferred may depend on sample size and on the context of research or application. Weighted sum scores could be particularly relevant in individual diagnostic settings. They are of similar reliability and validity as CFA-based scores and can be computed easily for each individual based on fixed circumplex weights without requiring a large sample size for preliminary CFA.

## Data Availability Statement

The raw data supporting the conclusions of this article will be made available by the authors, upon request without undue reservation.

## Ethics Statement

The studies involving human participants were reviewed and approved by the Ethics Board of the University of Bonn, Germany. Written informed consent from the participants' legal guardian/next of kin was not required to participate in this study in accordance with the national legislation and the institutional requirements.

## Author Contributions

ACW and VS carried out the study under the supervision of AB. ACW wrote the manuscript. All authors contributed to the revision of the manuscript and approve of its submission.

## Conflict of Interest

The authors declare that the research was conducted in the absence of any commercial or financial relationships that could be construed as a potential conflict of interest.

## Publisher's Note

All claims expressed in this article are solely those of the authors and do not necessarily represent those of their affiliated organizations, or those of the publisher, the editors and the reviewers. Any product that may be evaluated in this article, or claim that may be made by its manufacturer, is not guaranteed or endorsed by the publisher.

## Abbreviations

IIP: Inventory of Interpersonal Problems
SPMC: Circular Stochastic Process Model
MCFA: Confirmatory factor analysis with maximum-likelihood estimation
BCFA: Bayesian confirmatory factor analysis
TEFA: Exploratory factor analysis with subsequent target rotation toward the perfect circumplex
PSR: Potential scale reduction.
