# Supplementary material for: Bayesian and Maximum-Likelihood Modeling and Higher-Level Scores of Interpersonal Problems With Circumplex Structure
Source: Front Psychol. 2021 Oct 29;12:761378. doi: 10.3389/fpsyg.2021.761378 (PMC8586698; doi:10.3389/fpsyg.2021.761378)
Supplement: Supplementary file 1 [file Data_Sheet_1.PDF]

Supplementary Material

Figure S1

Trace plot of the potential scale reduction (PSR) for Bayesian confirmatory factor analysis based on larger and smaller prior variances

(A)

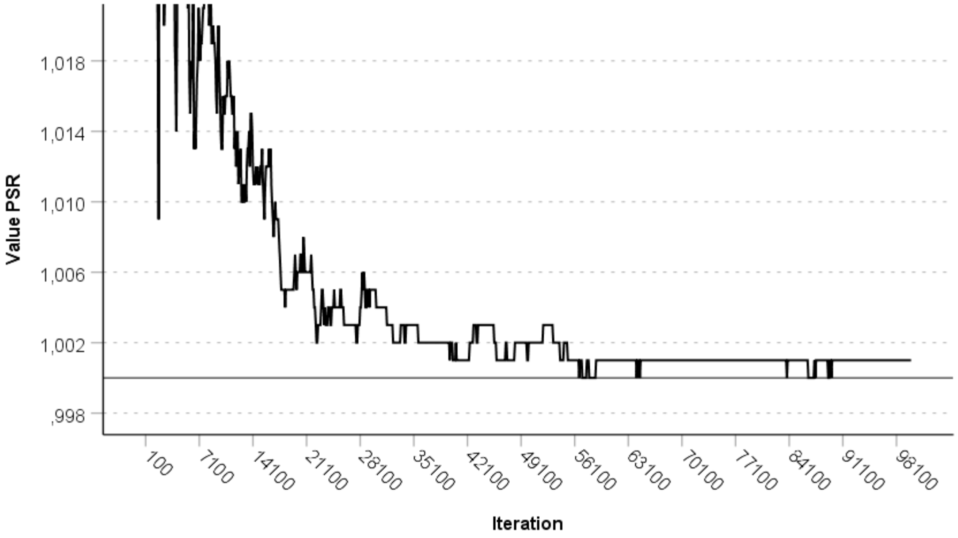

(B)

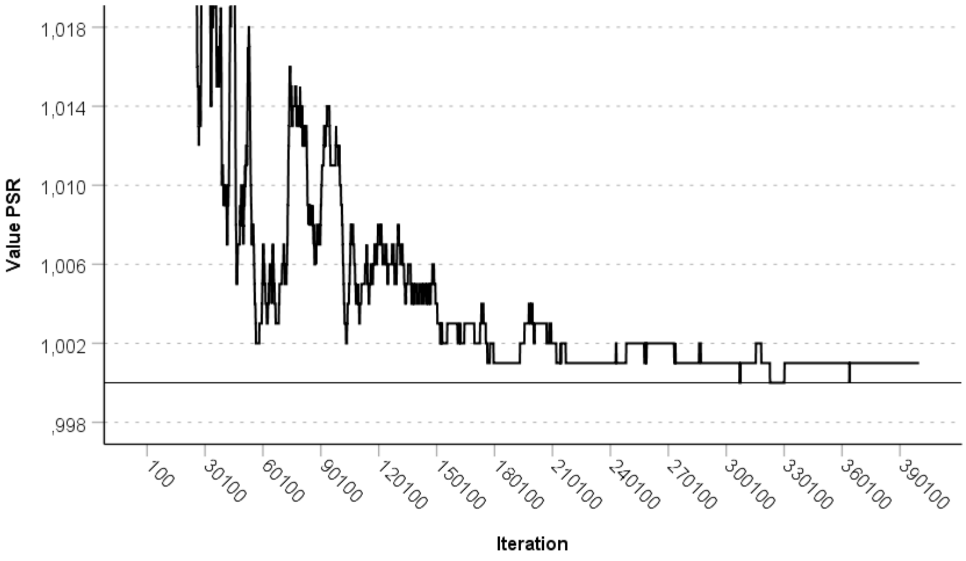

Note. Trace plot of the potential scale reduction (PSR) for 100,000 iterations and a prior variance of $\sigma^2 = 0.01$ (A) and for 400,000 iterations and a prior variance of $\sigma^2 = 0.1$ (B). There were two Markov chains, but we used only the second chain for the plot.

Supplementary Tables

Table S1

Standardized weights for the computation of higher-level scores for the Inventory of Interpersonal Problems

	MCFA regression scores			Weighted sum scores		
Scale	Dominance	Love	Distress	Dominance	Love	Distress
PA	.36	.03	.16	1	0	.13
BC	.38	-.26	.26	.71	-.71	.13
DE	.03	-.31	.17	0	-1	.13
FG	-.29	-.34	.21	-.71	-.71	.13
HI	-.34	-.04	.13	-1	0	.13
JK	-.35	.41	.27	-.71	.71	.13
LM	.14	.41	.23	0	1	.13
NO	.24	.18	.12	.71	.71	.13

Note. Higher-level scores for the sample in this study can be computed as sums of the standardized scale scores multiplied by the respective weights. MCFA regression scores were perfectly predicted by the eight scales and the weights ($R^2 = 1$).

Table S2

Model fit of an odd-even split file confirmatory factor analysis of the Inventory of Interpersonal Problems

Model	Sample/*N*	χ^2	*df*	*p/ppp*	*CFI*	Δ*CFI*	*RMSEA*	Δ*RMSEA*	*SRMR*	*BIC*	Δ*BIC*
MCFA	S1 / *N*=411	132.85	18	<.001	0.932	-0.012	0.131	0.013	0.085	7581.39	-191.45
	S2 / *N*=411	101.33	18	<.001	0.944		0.118		0.098	7772.84	
BCFA	S1 / *N*=411	3.42/54.34	-	.013	0.985	.002	0.095	-0.003	-	7716.20	-41.46
	S2 / *N*=411	5.14/56.48	-	.010	0.983		0.098		-	7757.66	

Note. MCFA = Maximum-likelihood confirmatory factor analysis; BCFA = Bayesian confirmatory factor analysis; S1 = Sample 1; S2 = Sample 2; *ppp* = posterior predictive *p*-value; *CFI* = Comparative Fit Index; Δ*CFI* = *CFI* S1 minus *CFI* S2; *RMSEA*= Root Mean Squared Error of Approximation; Δ*RMSEA* = *RMSEA* S1 minus *RMSEA* S2; *SRMR* = Standardized Root Mean Squared Residual; *BIC* = Bayesian Information Criterion; Δ*BIC* = *BIC* S1 minus *BIC* S2.
